# Provider-Initiated HIV Testing and Counseling: Increased Uptake in Two Public Community Health Centers in South Africa and Implications for Scale-Up

**DOI:** 10.1371/journal.pone.0027293

**Published:** 2011-11-17

**Authors:** Shona Dalal, Chung-won Lee, Thato Farirai, Allison Schilsky, Thurma Goldman, Janet Moore, Naomi N. Bock

**Affiliations:** 1 Epidemic Intelligence Service, Centers for Disease Control and Prevention, Atlanta, Georgia, United States of America; 2 Global AIDS Program, Centers for Disease Control and Prevention, Atlanta, Georgia, United States of America; 3 Global AIDS Program, Centers for Disease Control and Prevention, Pretoria, South Africa; Finnish Institute of Occupational Health, Finland

## Abstract

**Background:**

International guidance recommends the scale up of routinely recommended, offered, and delivered health care provider-initiated HIV testing and counseling (PITC) to increase the proportion of persons who know their HIV status. We compared HIV test uptake under PITC to provider-referral to voluntary counseling and testing (VCT referral) in two primary health centers in South Africa.

**Methods:**

Prior to introducing PITC, clinical providers were instructed to refer systematically selected study participants to VCT. After PITC and HIV rapid test training, providers were asked to recommend, offer and provide HIV testing to study participants during the clinical consultation. Participants were interviewed before and after their consultation to assess their HIV testing experiences.

**Results:**

HIV test uptake increased under PITC (OR 2.85, 95% CI 1.71, 4.76), and more patients felt providers answered their questions on HIV (104/141 [74%] versus 73/118 [62%] for VCT referral; p 0.04). After three months, only 4/106 (3.8%) HIV-positive patients had registered for onsite HIV treatment. Providers found PITC useful, but tested very few patients (range 0–15).

**Conclusion:**

PITC increased the uptake of HIV testing compared with referral to onsite VCT, and patients reported a positive response to PITC. However, providing universal PITC will require strong leadership to train and motivate providers, and interventions to link HIV-positive persons to HIV treatment centers.

## Introduction

Well into the third decade of the worldwide human immunodeficiency virus (HIV) epidemic, less than one-third of people in countries with generalized or emerging HIV epidemics know their HIV serostatus [1]. Access to HIV counseling and testing, an essential first step for prevention and HIV care and treatment service access, was constrained by a model for HIV testing developed decades ago in response to different circumstances: a disease with no treatment, lack of rapid testing to provide same day results, and concentration among persons already marginalized by illicit or stigmatized behaviors [2]. Most voluntary counseling and testing (VCT) sites were located in the community, not in health facilities, and any utilization was self-initiated. HIV testing was introduced into sub-Saharan African countries using this same VCT model [3]–[6].

The revised Policy Statement on HIV Testing published by the Joint United Nations Programme on HIV/AIDS (UNAIDS) and the World Health Organization (WHO) in 2004 emphasized the importance of knowledge of HIV status for expanding access to prevention, treatment, and care, and the importance of a serostatus-based approach to prevention has been further delineated [7], [8]. The recent results of a large multinational clinical trial indicating that earlier initiation of antiretroviral therapy among men and women infected with HIV reduced the risk of transmitting the virus to their sexual partners imparts even more urgency to the need for more widespread uptake of HIV testing and counseling [9].

The United States Centers for Disease Control and Prevention (CDC), WHO and UNAIDS have issued guidance recommending that any contact with the health care system should result in routinely recommended, offered, and delivered HIV testing initiated by health care providers (provider-initiated HIV testing and counseling or PITC) [10], [11]. The advantages of PITC over traditional client-initiated VCT are fourfold. First, being offered an HIV test by a clinical provider normalizes the test procedures similar to those for other diseases, and thus reduces stigma. Second, the focus on prevention information rather than individual risk reduction counseling in the PITC procedure, and the interaction with one provider rather than a minimum of two in VCT (a VCT counselor and an HIV testing nurse), dramatically decreases the time for obtaining an HIV test. Third, including HIV infection in any differential diagnosis in countries with generalized epidemics aids providers and HIV treatment programs with early detection of HIV infection. Lastly, offering HIV testing to a large proportion of patients in primary health care facilities substantially increases the pool of persons who know their status, enabling them to seek treatment if indicated, or to take precautions to remain uninfected. PITC in antenatal, tuberculosis, sexually transmitted infection clinics and inpatient wards has demonstrated increased uptake of testing [12]–[16]. Outpatient clinics are an additional important place to provide PITC. Nonetheless, incorporating PITC into busy, often understaffed, general outpatient clinics in resource-limited settings requires evaluation of its effectiveness and acceptance by both patients and staff.

South Africa has the highest number of persons living with HIV/AIDS (approximately 5.7 million) in the world today [17]. At the time of this study, eighteen per cent of adults between the ages of 15–49 were estimated to have HIV infection [17]; current estimates are the same [18]. The HIV testing standard in South Africa followed the VCT model. Although most VCT centers are physically located within community health centers (CHC), hospitals, and other health facilities, at the time of this study, the model was still a client-initiated one, rather than integrated into routine health services. Despite the availability of testing sites in most health facilities, the proportion of adults who were estimated to know their HIV status in 2005 was 30% [19]. This study was undertaken to inform South Africa's decision-making on implementing a different model to expand HIV testing and counseling.

We compared a PITC model, where providers routinely recommended and offered HIV testing to general adult outpatients, and provided the test to those who did not refuse, to one where providers referred outpatients to VCT (from here on referred to as the VCT referral model). The goals of this study were to: (1) determine whether the PITC model increased HIV testing among CHC outpatients as compared to the VCT referral model, (2) determine patients' experience and perceptions of HIV testing under the two models, and (3) evaluate health care provider acceptance and willingness to provide PITC to patients.

## Methods

### Setting

Study sites were selected with the Gauteng Province Department of Health based on type of clinic (government-operated CHC), average number of outpatients seen per day (to ensure sample size criteria and to evaluate PITC in a typical busy outpatient setting), and clinic administrators' willingness to participate. All government-operated CHCs provide all services free of charge and follow standardized procedures. Out of 30 CHCs in the province, 12 met the criteria for average number of patients, and of these, two large CHCs serving predominantly Black, low-income communities were designated by the National Department of Health to participate with their administrators' approval. One was located in Johannesburg, and the other in a rural township outside the city. Both facilities provide basic outpatient, labor and delivery, and HIV care and treatment services.

On average, approximately 500 adult outpatients seek care at the Johannesburg CHC daily, and approximately 300 at the rural one. During the study period there were seven part-time doctors and approximately 20 nurses seeing patients in the larger health center, and two doctors and 12 nurses in the smaller. Both CHCs had HIV treatment centers on site which provided free CD4 testing, cotrimoxazole for those not yet treatment eligible, and antiretroviral therapy for those eligible. CHC VCT centers were located roughly 10 meters from the outpatient consultation rooms in the same building.

### Study design

**Figure 1:**
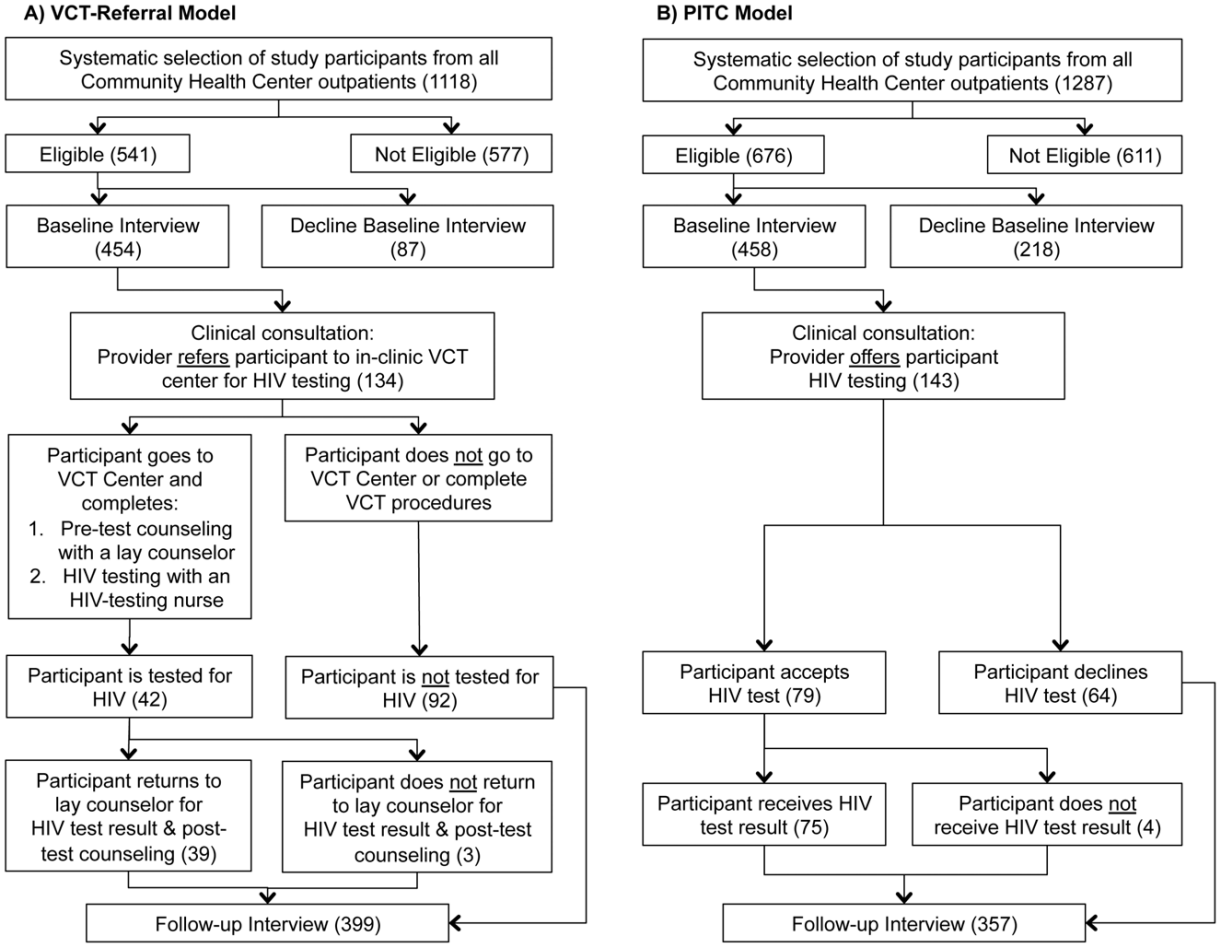
Participant sample sizes are given in parentheses. Participant enrollment algorithm and HIV testing procedures under the VCT referral (A), and PITC models (B).

We used a pre-intervention/post-intervention study design, with the pre-intervention VCT referral model serving as a control group to compare the effect of the post-intervention PITC model on HIV test acceptance. Figure 1 details each model. The intervention was training clinic nurses and physicians in conducting PITC and performing HIV rapid tests.

### Eligibility criteria

Patients were eligible for participation in the study if they were registered to be seen in the general adult outpatient clinic, between 18–49 years of age, competent to give informed consent (as determined by responses to two questions included in the consent procedure), a current resident of Gauteng province, and spoke English, Zulu, Sesotho, or Setswana (the four most common languages in the area). Pregnant women were excluded and referred to the antenatal care clinic to receive HIV counseling and testing in the context of prevention of mother to child transmission.

### Procedures

Each day systematic sampling was used to recruit general adult outpatients to participate in the study based on the queue number they received on entering the health center. Trained study interviewers first determined eligibility and willingness to participate among those selected, obtained informed consent, and then conducted structured face-to-face baseline interviews, all before the participant's clinical consultation. Participants were given a study identification card to present to the clinical provider when they entered the clinical consultation room. Participants were asked to return for short follow-up interviews at the end of their clinic visit to assess their experiences with HIV testing that day. Interview data were entered directly into hand-held computers.

#### VCT referral model

Providers were instructed to provide a brief statement about the importance of knowing one's HIV status to all study participants, and to refer them to the on-site VCT center during two weeks in July 2007. Standard VCT procedures included the following sequence (i) approximately 20-minutes pre-test counseling by a lay counselor, (ii) HIV rapid testing performed by a designated nurse in a serial format as per the South African national standard and (iii) post-test counseling by the lay counselor.

#### PITC model

After completing data collection for the evaluation of the VCT referral model, we trained providers in PITC and HIV rapid testing. We then allowed two weeks of observed PITC implementation without data collection to allow providers to familiarize themselves with the procedures and for problem-solving. For the PITC model evaluation, we instructed providers to recommend and offer HIV testing to study participants, and to provide testing to those who did not decline during the clinical consultation over two weeks in August 2007. Providers were also asked to offer HIV tests to as many additional non-study patients per day as possible.

During implementation of both the VCT referral and PITC models, persons identified as HIV-positive were referred by their provider or counselor to the onsite HIV treatment clinic. Three months after implementation, we reviewed the onsite HIV treatment clinic records to determine the proportion of patients who reported having tested HIV-positive during the study, who were documented to have received follow up HIV care at the same CHC.

The study protocol was approved by the South African Medical Association Research and Ethics Committee and the CDC institutional review board. Written informed consent was obtained twice from all study participants, once for the baseline interview and a second time for the follow-up interview.

### Provider attitudes

Providers completed brief, anonymous questionnaires on knowledge and attitudes to HIV testing before the study started, after being trained in PITC, and again after PITC model implementation. On conclusion of the study, we held informational interviews with providers to discuss the PITC model successes, challenges, feasibility and impact on their workload. These discussions were led by an experienced facilitator, and questions and responses were documented by two recorders during each session. After merging the two recorders' reports, a content analysis was conducted on the resulting transcript to identify common themes.

### Statistical analysis

Data were analyzed using SAS software (version 9.1, SAS Institute, Cary, NC). For bivariate analysis of categorical variables, we compared proportions using chi-square or Fisher's exact tests. For continuous variables, we compared means using the student's t test and medians using the Wilcoxon rank-sum test. Multiple logistic regression was used to calculate odds ratios to identify factors associated with getting tested for HIV infection. The logistic regression models included key pre-test variables and potential confounding variables, which were selected by investigators based on subject matter knowledge.

## Results

### Participation

Eligibility surveys were completed by 1118 outpatients during VCT referral model and 1287 during PITC model implementation. Of these, 51% were eligible (541 during the VCT referral model and 676 during the PITC model). Of eligible outpatients, 454/541 (84%) during the VCT referral, and 458/676 (68%) during the PITC model implementation consented to participate and completed baseline questionnaires. The most frequently cited reasons for refusing participation were not having enough time (59%), feeling too ill (16%), and not being interested in participating (14%). Of those who completed baseline questionnaires, 756 (83%) returned for a follow-up interview (399/454 [88%] during VCT referral and 357/458 [78%] during PITC; p<0.0001). The difference in participation was almost entirely accounted for in one clinic, which had a drop in staffing levels during the PITC evaluation.

### Study population

Comparisons of self-reported baseline participant characteristics by the model of HIV testing they received are shown in Table 1. The median age was 33 and 574/912 (63%) of participants were female. The two groups were similar except for three variables which showed statistically significant but small differences between the study participants in the two models.

**Table 1:** Study participant characteristics by HIV testing model.*

Characteristics	VCT ReferralN = 454	PITCN = 458	TotalN = 912	p-value
Clinic: CHC A	263 (58%)	243 (53%)	506 (56%)	0.14
Sex: Female	287 (63%)	287 (63%)	574 (63%)	0.86
Age				0.86
18–29 years	169 (38%)	179 (39%)	348 (38%)	
30–39 years	146 (33%)	147 (32%)	293 (32%)	
40–49 years	134 (30%)	130 (29%)	264 (29%)	
Education				0.44
≤4 years	36 (8%)	34 (7%)	70 (8%)	
5–8 years	95 (21%)	100 (22%)	199 (21%)	
9–12 years	289 (64%)	276 (60%)	577 (62%)	
13≤years	32 (7%)	47 (10%)	80 (9%)	
Marital Status				0.35
Married	83 (18%)	89 (19%)	172 (19%)	
Separated, divorced, widowed	74 (16%)	62 (14%)	136 (15%)	
Never married	295 (65%)	306 (67%)	601 (66%)	
Currently living with sex partner	91 (25%)	99 (27%)	190 (26%)	0.49
Currently employed	221 (49%)	185 (40%)	406 (45%)	0.01
Primary care-giver for child <18 years	299 (66%)	270 (60%)	569 (63%)	0.02
Ever tried injecting drug	2 (0.4%)	3 (0.6%)	5 (0.5%)	1.00
Men: Ever had sex with a man	2/165 (1%)	1/166 (1%)	3 (1%)	0.62
Ever had an STI	96 (21%)	78 (17%)	174 (19%)	0.11
Median number of times visited medical facility in last 12 months (IQR)	3 (2–6)	2 (1–4)	3 (1–5)	<0.0001
Concurrent sex in past 12 months**	69 (19%)	62 (17%)	131 (18%)	0.62
Ever tested for HIV	275 (61%)	252 (56%)	527 (59%)	0.13

*Number in strata may not equal total N due to some missing values. All percentages may not add up to 100% due to rounding.

**Answered yes to either: *“At the time you were having a sexual relationship with this regular partner, did you have sex with other people?”* or *“At the time you having a sexual relationship with this regular partner, was your partner having sex with other people?”*.

Abbreviations: VCT, voluntary counseling and testing; PITC, provider-initiated HIV testing and counseling, CHC A, community health center A; STI, sexually transmitted infection; IQR, interquartile range; HIV, human immunodeficiency virus.

### HIV Test Acceptance

The proportion of participants who reported being referred to VCT (134/399 [34%]) was slightly lower, but not statistically different, than those reporting being offered PITC (143/357 [40%], p = 0.06). In unadjusted analyses, significantly more participants in the PITC model reported that they accepted HIV testing as compared to those who reported following the referral to VCT and getting tested (79/143 [55%] versus 42/134 [31%]; odds ratio (OR) 2.70, 95% confidence interval (CI) 1.65, 4.42) (Table 2). The majority of participants reported receiving their HIV test results, 39/42 (93%) in the VCT referral and 75/79 (95%) in PITC model.

**Table 2:** Multiple logistic regression of selected pre-test factors with accepting an HIV test among 277 outpatients referred to VCT or offered a test by their provider (PITC).

Factor	Proportion accepted HIV test (%)	Crude OR for Tested (95% CI)	Adjusted OR (95% CI)*
Testing model			
PITC	79/143 (55)	2.70 (1.65–4.42)	2.85 (1.71–4.76)
VCT referral	42/134 (31)	Referent	
Clinic			
CHC A	76/168 (45)	1.18 (0.72–1.91)	1.26 (0.73–2.17)
CHC B	45/109 (41)	Referent	
Age			
18–29 years	43/105 (41)	Referent	
30–39 years	40/94 (43)	1.07 (0.61–1.88)	0.96 (0.52–1.75)
40–49 years	37/75 (49)	1.40 (0.77–2.55)	1.18 (0.60–2.32)
Sex			
Female	81/175 (46)	1.34 (0.81–2.19)	1.42 (0.82–2.45)
Male	40/102 (39)	Referent	
Had previous HIV test			
Yes	79/163 (48)	1.73 (1.05–2.86)	1.70 (0.98–2.94)
No	38/108 (35)	Referent	
Possible to get confidential HIV test in their community			
Yes	104/228 (46)	2.18 (1.01–4.73)	2.09 (0.90–4.91)
No	10/36 (28)	Referent	
Ever thought themselves infected with HIV			
Yes	45/115 (39)	0.73 (0.46–1.23)	0.80 (0.47–1.37)
No	70/152 (46)	Referent	
Ever had a STI			
Yes	26/53 (49)	1.33 (0.73–2.42)	1.55 (0.81–2.97)
No	93/221 (42)	Referent	
Ever forced or coerced into sex			
Yes	22/38 (58)	2.02 (1.01–4.05)	2.06 (0.97–4.39)
No	92/227 (41)	Referent	
Concurrent sex in past 12 months**			
Yes	14/36 (39)	0.81 (0.39–1.69)	0.85 (0.38–1.87)
No	77/175 (44)	Referent	
Heard ART available in Gauteng Province			
Yes	80/168 (48)	1.50 (0.82–2.74)	1.53 (0.79–2.96)
No	23/61 (38)	Referent	

*Adjusted for age, sex, education, clinic, and testing model.

**Answered yes to either: *“At the time you were having a sexual relationship with this regular partner, did you have sex with other people?”* or *“At the time you having a sexual relationship with this regular partner, was your partner having sex with other people?”*.

Number in strata may not equal total N (277) due to some non-applicable questions and some missing values. No more than 11% of responses were missing. All percentages may not add up to 100% due to rounding.

Abbreviations: VCT: Voluntary HIV testing and counseling, PITC: Provider-initiated HIV testing and counseling, HIV: Human immunodeficiency virus, STI: Sexually transmitted infection, OR: Odds ratio, CI: 95% Confidence interval, CHC: community health center; ART, antiretroviral therapy.

Factors reported at baseline that were associated with test acceptance included having been previously tested for HIV (OR 1.73, CI 1.05, 2.86), believing that it was possible to get a confidential HIV test in the community (OR 2.18, CI 1.01, 4.73), and ever being forced or coerced into sex (OR 2.02, CI 1.01, 4.05) (Table 2). In multiple logistic regression analyses that adjusted for age, sex, education, and clinic, those offered HIV testing under the PITC model were still more likely to accept a test (adjusted odds ratio (aOR) of 2.85, (CI 1.71, 4.76)) (Table 2). Having had a previous HIV test showed some evidence of an association with increased HIV test acceptance (aOR 1.70, CI 0.98–2.94), as did ever being forced or coerced into sex (aOR 2.06, CI 0.97–4.39). Of the factors associated with the acceptance of an HIV test, none showed a differential association (i.e. interaction) with model of testing (results not shown).

The most frequently cited reasons participants gave for declining an HIV test were that they were uncomfortable or afraid of the HIV test (31%), they did not feel the need to be tested (19%), they were tested in the past with an HIV-positive result (11%), or they were in a hurry (7%).

At follow-up interviews, significantly more participants reported that their provider answered their questions on HIV under the PITC model as compared to the VCT referral model (104/141 [74%] versus 73/118 [62%]; p = 0.04) (Table 3). Otherwise, there were no significant differences in perceptions of the two models.

**Table 3:** Post-test patient experience and perceptions of HIV testing among those referred to VCT or offered a test by their provider (PITC), by testing model.*

Patient Experience	VCT Referral	PITC	
*Answered “yes” to the following questions*	(N = 134)	(N = 143)	p-value
Questions on HIV were answered by provider**	73/118 (62%)	104/141 (74%)	0.04
Could say no to HIV test**	52/119 (44%)	64/143 (45%)	0.86
Had enough time to discuss HIV test results***	35/39 (90%)	71/75 (95%)	0.44
Will tell someone about their HIV test***	31/39 (79%)	68/75 (91%)	0.09
HIV test should be offered with same model at community health centers	116/119 (97%)	142/143 (99%)	0.33
Others would test for HIV if offered a test with same model they received	100/115 (87%)	116/140 (83%)	0.37
Fear of being offered an HIV test by provider would not prevent patients from coming to CHC	–	98/143 (69%)	–
Patients who test for HIV at CHC would not face problems at home or in the community	81/131 (62%)	82/143 (57%)	0.45
Was treated with respect at the CHC	127/134 (95%)	133/143 (93%)	0.54

*Numbers in strata may differ from total N due to missing values as some participants chose not to answer a question.

**Variables associated with HIV test uptake.

***Asked only of participants who agreed to undergo HIV testing.

Abbreviations: VCT: Voluntary HIV testing and counseling, PITC: Provider-initiated HIV testing and counseling, HIV: Human immunodeficiency virus, CHC: community health center.

### HIV prevalence among study participants

During VCT referral model implementation, 9 participants out of 42 (21%) tested HIV positive, as did 19 of 79 (24%) participants during the PITC model implementation (chi square 0.106, p 0.74).

### Linkage of those testing HIV positive to the HIV treatment clinic

Providers trained in PITC tested an additional 229 non-study patients during the PITC model implementation, 80 (35%) of whom tested HIV positive. All HIV positive patients were referred to the HIV treatment clinic. Three months after study and non-study patients' positive HIV test, four (3.8%) had registered at the onsite HIV treatment clinic. This included 1 of the 7 patients who tested HIV positive during the VCT referral model for whom we had data, and 3 of 99 patients who tested HIV positive during the PITC model (19 study participants and 80 other patients undergoing PITC during the same time period). Due to a recording error, treatment follow-up information was not available for 2 HIV positive individuals from the VCT referral model.

### Provider feedback on PITC

Providers tested a mean of 2 patients per day (range 0–15) during the PITC model implementation. All 23 providers who offered HIV tests using the PITC model thought that it was important and useful for patient care; 96% thought patients may be more likely to get a test if it was offered by their clinical provider.

In informational interviews and discussions, providers identified the following challenges to PITC implementation: PITC significantly adds to an already excessive workload; shared consulting rooms limit providers' ability to ensure confidentiality for patients during the process; providing PITC on-the-job training for new staff will be difficult; and ensuring an adequate supply of HIV testing consumables will be challenging. Despite these barriers, providers reported that PITC empowered them to better care for their patients, and reported that patients appreciated that HIV testing was provided in the same consultation room with no additional wait-time required.

## Discussion

Provider-initiated HIV testing and counseling among adult general outpatients in two high-volume primary care clinics in Gauteng Province, South Africa resulted in a 2.85 fold increase in odds of HIV test acceptance as compared to provider referral to onsite VCT services in the same clinics. Patients' reported experiences of the two models were similar and positive, though significantly more patients reported that their providers answered their questions about HIV in the PITC model. The median age of study participants was 33 years, 63% were women, and 66% had never been married; thus clinic patients were representative of a population with high HIV prevalence in South Africa. Among study participants in both the PITC and VCT referral model, more than one in five among those tested was HIV positive. In both models, documented linkage to HIV care among those who tested positive was extremely low. Providers expressed appreciation of the value of PITC in answers to written questions and in discussions, indicating that it assisted them with patient care; however they tested only a small percentage of their patients.

Among those who declined testing with either model, almost one-third (31%) refused because they were uncomfortable or afraid of an HIV test and 19% reported not feeling the need to be tested. These reasons are similar to the published literature, and indicate that continuing widespread fear of HIV testing must be addressed [20], [21], [22]. In both the VCT referral and PITC models, over half the participants reported that they did not feel they could decline the test. These findings are unclear as many of these same participants did in fact refuse testing. Nonetheless, guidance and ongoing supervision must be provided to health care providers implementing PITC to ensure patients can opt out of testing [11]. Evidence from our study suggests that test uptake was associated with having had a previous HIV test and ever being forced or coerced into sex, but not with any other reported risk behaviors for HIV acquisition.

One of the strengths of our study is that during PITC model implementation, the providers themselves offered and performed PITC as part of the general outpatient visit. Increased acceptance of HIV testing by general outpatients offered PITC has been previously reported in Zambia and South Africa, but in both those studies lay counselors rather than clinicians offered and provided the HIV testing and counseling in the outpatient department [22], [23]. Similar rates of increased testing were seen in those studies as the 1.8 fold increase in ours: in Zambia, the addition of lay counselor-conducted PITC to referral to VCT doubled the number tested for HIV in 9 primary care clinics compared with referral to VCT alone. In Durban, South Africa, acceptance of testing was 1.5 times higher with PITC conducted by lay counselors compared with referral to VCT by clinicians. Clinicians themselves performing routine HIV testing, and associated increased testing uptake, has been reported in antenatal, TB, and STI clinics in southern Africa [12]–[16], but to our knowledge this is the first report of clinicians implementing PITC in general outpatient clinics with very high daily patient volumes.

Another strength is that we assessed provider and patient attitudes and perceptions of PITC during its implementation, and compared and contrasted these with HIV test acceptance results. Contradictory findings included that providers expressed appreciation for the value of PITC for improving patient care, but tested very few patients. Confirmatory findings included that patients who reported that it was possible to get a confidential HIV test in their community were more likely to accept testing. These findings from provider and patient surveys can inform program improvements.

Furthermore, we followed participants beyond uptake of HIV testing to determine the linkage of those who tested positive to HIV care and treatment services. Many studies have reported an increase in HIV test acceptance with PITC; few have documented whether the HIV-infected persons identified benefited from their known status by accessing HIV clinical services [24]. We found only four percent of HIV-positive patients had a registered visit to the onsite HIV treatment clinic three months after their test result. A priority area for further research is to investigate the reasons for this lack of follow-up.

There were several limitations to this study. First, the study design, a pre-intervention/post-intervention evaluation, lacks the rigor of a randomized controlled trial. The two health centers were typical of health centers in South Africa, but may not be representative of other types of health facilities. There was a decline in the rate of participation and follow up interviews during the PITC data collection period, which was observed in one of the two clinics. This difference was likely due to a drop in staffing at that health center during PITC implementation, so that many patients left without being seen by a provider, including enrolled participants who had completed baseline questionnaires. It is unlikely that this affected HIV test acceptance at the clinic. The use of self-reported data from participants carries the inherent possibility of social desirability bias. However, it seems unlikely there would be differential reporting between the participants in the two models of testing. Furthermore, self-reported HIV status has been shown to have similar validity to other self-reported variables [25]. Lastly, the follow-up of patients at the HIV treatment clinic at the CHC where they were tested may not be an indication of an individual accessing care. Patients may have chosen to go to another HIV treatment clinic for reasons of convenience or perceived confidentiality.

Several programmatic recommendations follow from our study results. First, regarding the low rate of testing by providers during the PITC model implementation. Provider performance even in high-volume clinics can be influenced by strong leadership from all administrative levels of the health system to create a sense of professional responsibility for improving patients' knowledge of their HIV status. Furthermore, in settings such as South Africa where overall one in every five adults is HIV-infected, determining HIV status should be considered a necessary part of a differential diagnosis for any acute medical conditions. Since this study was completed, the South African Minister of Health has endorsed PITC, which should lead to changed expectations of providers' performance [26].

Under current staffing conditions, it will be very difficult to achieve universal HIV testing through PITC in South African community health centers. To do so, for a CHC serving 400 patients a day with 12 providers offering PITC (the averages from our study), each provider would need to test 33 patients per day on average (results not shown). If however, HIV testing was recommended once per year for those first testing negative, that number could fall to 11, as patients reported visiting the same health center a median of three times per year. Under these conditions, encouraging providers to test 6 patients per day on average would ensure that roughly 50% of outpatients would be offered an HIV test in a given year.

A second essential area for long-term prevention programming in addition to increasing testing rates, is determining the barriers to successfully linking patients who test HIV positive to treatment services, and implementing interventions to overcome these barriers at the structural and individual level. For example, Gauteng Province is instituting a patient locater system, which will include all government HIV care programs, so that patient access to care can be tracked across facilities. Determining the effectiveness of this system in improving retention will be key.

Finally, using a parallel rather than a serial HIV rapid testing algorithm would reduce the time necessary for processing HIV tests, and improve the efficiency of both models of HIV testing. Recent legislation in South Africa has for the first time allowed lay counselors to conduct HIV rapid testing, which will streamline the VCT referral model.

In conclusion, PITC increased the uptake of HIV testing compared with referral to onsite VCT in two government-operated, free of charge, community health centers in South Africa, and patients reported a positive response to PITC. The proportion of patients who were tested was low in both models of HIV testing, a concern in a country with high prevalence of HIV infection; among those tested, the proportion of patients who tested HIV positive was high. PITC allowed health care providers to identify many HIV infected general outpatients, but some key challenges should be addressed as it is scaled up to complement existing VCT services. Health facilities implementing PITC in the future will benefit from regional and facility-level PITC implementation plans including the development of training schedules, optimization of clinic flow and floor plans to ensure patient confidentiality, and administrative support to supervise and motivate health care providers. Finally, strengthening referral systems within and between health facilities to ensure that patients are effectively linked to treatment and prevention services will be vital to ensuring successful patient and programmatic outcomes.
